# Infection of endemic chub *Squalius tenellus* with the intestinal tapeworm *Caryophyllaeus brachycollis* (Cestoda): histopathology and ultrastructural surveys

**DOI:** 10.1017/S0031182023001233

**Published:** 2024-02

**Authors:** Bahram Sayyaf Dezfuli, Emanuela Franchella, Giovanni Bernacchia, Morena De Bastiani, Francesca Lorenzoni, Antonella Carosi, Massimo Lorenzoni, Giampaolo Bosi

**Affiliations:** 1 Department of Life Sciences and Biotechnology, University of Ferrara, St. Borsari 46, 44121 Ferrara, Italy; 2 Department of Chemistry, Biology, and Biotechnology, University of Perugia, St. Elce di sotto 5, 06123 Perugia, Italy; 3 Department of Veterinary Medicine and Animal Science, University of Milan, St. of University 6, 26900, Lodi, Italy

**Keywords:** fish innate immune cells, histopathology, mucous cells, rodlet cells, tapeworm, ultrastructure

## Abstract

The endemic chub *Squalius tenellus* (Heckel, 1843) was introduced more than 100 years ago to Lake Blidinje (Bosnia-Herzegovina). Only 1 species of enteric helminth was found in a sample of 35 chubs, the tapeworm *Caryophyllaeus brachycollis* (Janiszewska, 1953). The paper includes histopathological investigation with identification of innate immune cells involved in host reaction and molecular data allowed correct designation of the cestode species. Of 35 specimens of chub examined, 21 (60%) harboured individuals of *C. brachycollis* and a total of 1619 tapeworms were counted, the intensity of infection ranged from 1 to 390 worms per fish (46.2 ± 15.3, mean ± s.e.). Histopathological and ultrastructural investigations showed strict contact between the worm's body and the epithelia and increase in the number of mucous cells, rodlet cells among the epithelial cells. Within the tunica propria-submucosa, beneath the site of scolex attachment, numerous neutrophils and mast cells were noticed. This is the first study of the occurrence of *C. brachycollis* in chub from Lake Blidinje and on the response of the innate immune cells of *S. tenellus* to this tapeworm. Interestingly, in 3 very heavily infected chubs, perforation of the intestinal wall was documented; this is uncommon among cestodes which use fish as a definitive host.

## Graphical abstract

**Figure:**
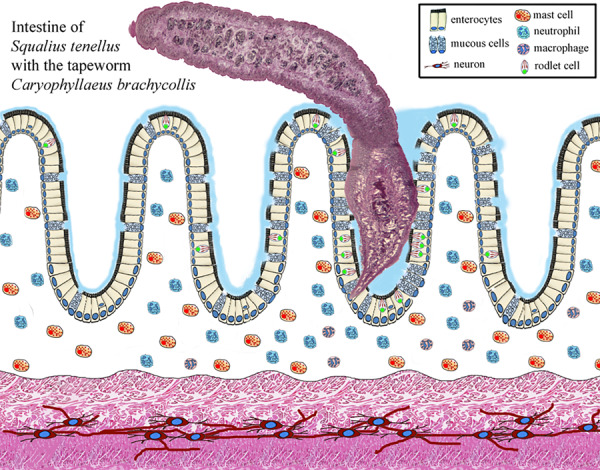

## Introduction

Chub *Squalius tenellus* (Heckel, 1843) most likely was introduced to the Lake Blidinje in a homonym Blidinje Natural Park more than 100 years ago (Buj *et al*., 2020). The lake is glacial type and the Blidinje Natural Park is a protected landscape category (IUCN V) situated in south of Bosnia-Herzegovina Federation. Our investigation revealed massive infection of chub with tapeworm *Caryophyllaeus brachycollis* (Janiszewska, 1953) and for this parasite this is the first study on type-host (*S. tenellus*) and type-locality (Lake Blidinje, Bosnia-Herzegovina). There is dearth of information on parasites of *S. tenellus*; we found 1 single record on occurrence of a monogenean in gills of this chub in Bosnia-Herzegovina Federation (Benovics *et al*., 2023). Molecular phylogenetic studies on fish tapeworms of the genus *Caryophyllaeus* Gmelin, 1790 (Cestoda: Caryophyllidea), parasites of cyprinid fishes in the Palaearctic region, have showed unexpected phenotypic plasticity that seems to be related to definitive hosts (Barčák *et al*., 2014). For example, *C. brachycollis* has 2 morphotypes: morphotype 1 from barbels (*Barbus* spp. including the type-host *Barbus barbus* and chubs (*Squalius* spp.)) and morphotype 2 from breams (*Abramis* spp., *Ballerus* spp. and *Blicca* spp.) (Barčák *et al*., 2014).

Most individual fish in wild and farmed populations can be infected with parasites. Fish intestines can harbour protozoans, myxozoans and helminths (Sayyaf Dezfuli *et al*., 2021*a*). Helminths, a general term meaning worm, are among the most important groups of fish metazoan parasites. Parasitic Platyhelminthes comprise 3 classes: Monogenea, Trematoda (flukes) and Cestoda (tapeworms) (Gibson *et al*., 2014). Cestoda, or tapeworm, constitutes a large class of the Platyhelminthes with about 5000 species of which almost 500 species infect marine and freshwater fishes (Scholz *et al*., 2021). The extent of damage caused by tapeworms is generally due to the intensity of infection and depth of penetration into the host tissue; in fact, the relationship of the extent of pathology and scolex morphology in 15 species of caryophyllid cestodes was published in Mackiewicz *et al*. (1972) and a recent review of Scholz *et al*. (2021) provides new information on tapeworms as pathogens in fish. Many cestodes and trematodes do not provoke severe damage to the fish alimentary canal, inducing only destruction of the superficial layer of the intestine at the point of their attachment organs (Sayyaf Dezfuli *et al*., 2021*a*). Very seldom, cestodes penetrate more deeply, approaching the muscle layer and inducing destruction of the intestinal architecture (Mackiewicz *et al*., 1972; Molnár *et al*., 2003; Dezfuli *et al*., 2011). Herein, perforation of the intestinal wall was documented in 3 very heavily infected *S. tenellus*.

Enteric helminth infections commonly incite inflammation of the digestive tract and in several records it was documented that the occurrence of a parasite within a host can induce the formation and/or recruitment of various inflammatory cells, at the site of infection (Molnár *et al*., 2003; Sayyaf Dezfuli *et al*., 2021*a*; Scholz *et al*., 2021). Some of most common cell types associated with enteric parasite infections in fish are mast cells also known as eosinophilic granular cells (MCs) (Reite and Evensen, 2006; Sayyaf Dezfuli *et al*., 2021*a*), rodlet cells (RCs) (Sayyaf Dezfuli *et al*., 2022, 2023*a*, 2023*b*), neutrophils (Dezfuli *et al*., 2011) and mucous cells (Bosi *et al*., 2017, 2022).

The main purpose of our investigation was to document the inflammatory response of *S. tenellus* to the tapeworm *C. brachycollis*. We utilized histology and ultrastructural analysis to evaluate the occurrence and nature of the immune cells involved in defence of chub intestine against a harmful cestode. This is the first histopathological study on *S. tenellus* from Lake Blidinje infected with a tapeworm. Indeed, to avoid unreliable identification based only on morphological features of parasite species encountered in *S. tenellus*, this investigation provides molecular data which allowed correct designation of the tapeworm species.

## Materials and methods

In 2 occasions, May and July 2023, a subpopulation of 35 specimens of *S. tenellus* with total length (25.47 ± 1.01 cm, mean ± s.e.) and weight (235.6 ± 26.4 g, mean ± s.e.) were collected from Lake Blidinje in Blidinje Natural Park in southern Bosnia-Herzegovina Federation (43°36′25″N, 17°29′48″E). The fish sampling was carried out in a semi-quantitative way using gill nets of 2 mesh sizes (24 and 40 mm). After sampling, on field fish were anesthetized using MS222 (125 mg L^−1^, tricaine methanesulphonate, Sandoz, Basel, Switzerland) and weighed, measured and sexed (18 males, 17 females); once euthanized, the spinal cords were severed before the fish were dissected ventrally. Upon dissection, body cavity and visceral organs were examined in search of tapeworms; then, the alimentary canal was removed, opened longitudinally and position and number of worms were registered. Pieces of infected–uninfected intestine measuring up to 15 × 15 mm^2^ in size were excised and fixed in 10% neutral buffered formalin for 24 h. Thereafter, the samples were dehydrated through an alcohol series and then paraffin wax-embedded using a Shandon Citadel 2000 tissue processor. Multiple sections of 7 *μ*m were taken from each tissue block, stained with Alcian blue (AB) or haematoxylin and eosin and/or Giemsa and examined and photographed using a Nikon Microscope ECLIPSE 80i. Pieces of intestine of uninfected fish for comparative purpose were excised and fixed in 10% neutral buffered formalin for 24 h and proceeded as mentioned above. Multiple histological sections were taken from each tissue block, examined and photographed using an optical microscope (Nikon Eclipse 80i; Nikon, Tokyo, Japan).

For transmission electron microscopy (TEM), 140 pieces of 7 × 7 mm^2^ of 21 infected and 10 uninfected intestines were fixed in chilled 2.5% glutaraldehyde in 0.1 m sodium cacodylate buffer for 3 h. The fixed tissues were then post-fixed in 1% osmium tetroxide for 2 h and then rinsed and stored in 0.1 m sodium cacodylate buffer containing 6% sucrose for 12 h. Thereafter, the tissue pieces were dehydrated through a graded acetone series and embedded in epoxy resin (Durcupan ACM, Fluka, Buchs, Switzerland). Semi-thin sections (1.5 *μ*m) were cut on a Reichert Om U2 ultramicrotome and stained with toluidine blue. Ultra-thin sections (90 nm) were stained with 4% uranyl acetate solution in 50% ethanol and Reynolds’ lead citrate and then examined using a Talos L120C transmission electron microscope.

### Molecular characterization of parasite species

Genomic DNA was extracted from 3 tapeworms isolated from the intestine of *S. tenellus*; alive parasites were stored in absolute ethanol, using the NucleoSpin DNA Insect kit (Macherey-Nagel, Dűren, Germany), quantified by using a spectrophotometer (Bio-Spec Nano, Shimadzu, Milan, Italy) and analysed by gel electrophoresis. Polymerase chain reaction (PCR) amplifications were performed in accordance with Bazsalovicsová *et al*. (2014) using the same primers to amplify and sequence fragments from the mitochondrial gene cytochrome c oxidase subunit I (cox1, 657 bp) and from the large subunit of the nuclear ribosomal RNA (lsrDNA, 1578 bp) (Bazsalovicsová *et al*., 2014). PCR fragments were sequenced at BMR Genomics (Padova, Italy) using both amplification primers, while internal primers LSU and 1500 were also used in the case of lsrDNA. DNA sequences were then compared to GenBank accessions using BLAST software.

## Results

Sequence analyses for both DNA PCR fragments allowed us to identify the specimen as *C. brachycollis*, with a Ha1 *cox* haplotype. This haplotype was found by Bazsalovicsová *et al*. (2014) for tapeworms encountered in *Squalius cephalus* and *Barbus cyclolepis* specimens collected in Slovakia and Bulgaria.

**Figure 1.**
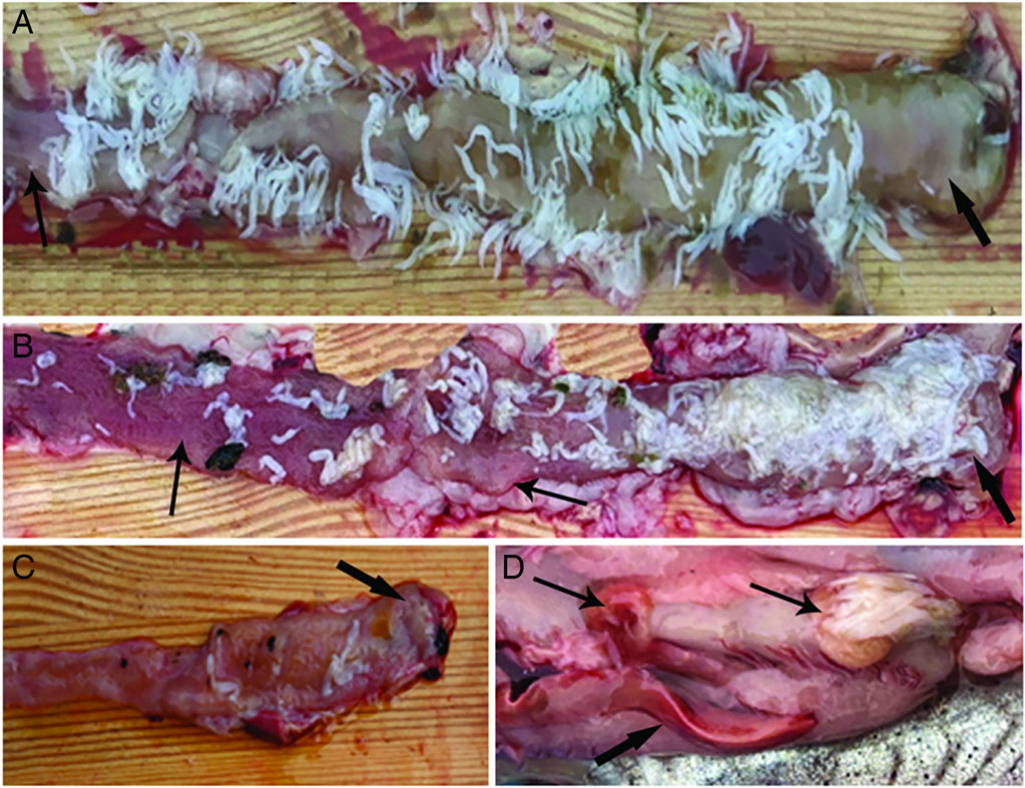
Photos after necropsy of *Squalius tenellus*. (A) Heavy infection of chub intestine due to *Caryophyllaeus brachycollis*; in some points the parasites occurred as cluster, oesophagus (thick arrow) and rectum (arrow) were less infected regions. (B) High infection of the anterior intestine; note the presence of numerous *C. brachycollis* also in oesophagus (thick arrow), in this host middle intestine and rectum were less parasitized regions (arrows). (C) Photo shows anterior intestine with very few tapeworms; arrow shows oesophagus. (D) Image of 1 very heavily infected chub during necropsy; cluster of worms perforated the intestine in 2 points (arrows); note extrusion of several strobilae on right side of the photo; reddish normal colour of liver (thick arrow) is appreciable.

Twenty-one (13 males, 8 females) of 35 chubs (60%) were infected with *C. brachycollis*, a total of 1619 tapeworms were registered, the intensity of infection was 1–390 worms per host (46.2 ± 15.3) (mean ± s.e.), in 8 fish with over 80 tapeworms, all the regions of the alimentary canal were parasitized and a density of 5 worms per cm^2^ was common (Fig. 1A and B). In the rest of parasitized fish (13), anterior and middle intestines were the most infected regions. Figure 1C shows the anterior intestine with a very few *C. brachycollis*. During necropsy, in 3 very heavily infected chubs, perforation of the intestines was noticed with part of long strobilae of the worms visible from the hole (Fig. 1D); indeed, in this group of hosts, the presence of several free worms in body cavity was noticed. *In situ*, an excessive yellowish mucus/catarrh was observed around the worms, which in histological sections appeared as a thick, adherent blanket of mucus that gave an intense positive signal when stained with AB (see further). The worms occurred either singularly (Fig. 1B and C) or more frequently in cluster (Fig. 1A and B).

**Figure 2.**
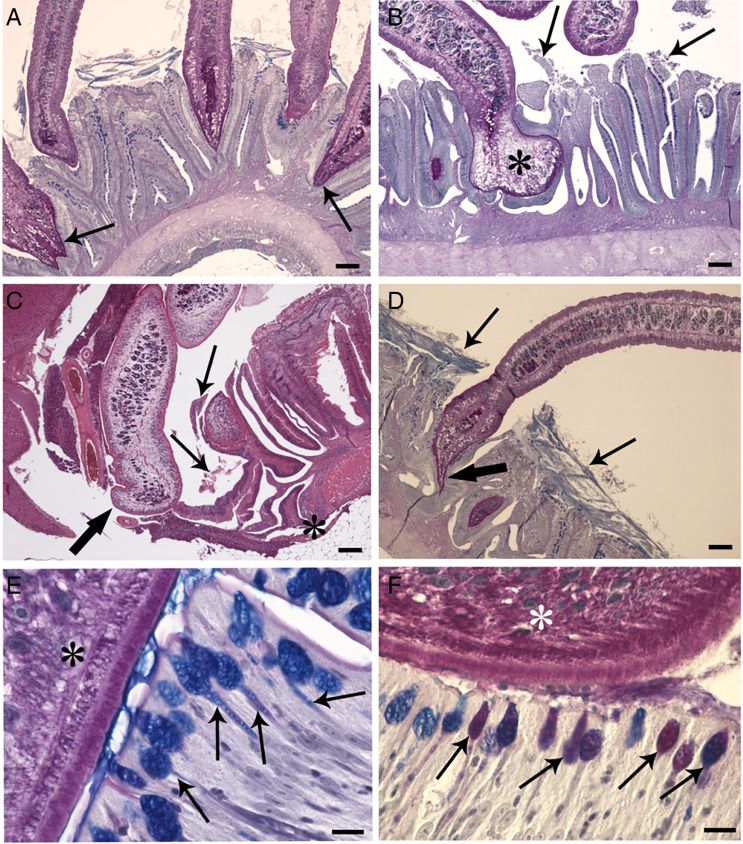
Histological sections of infected intestine of *S. tenellus.* (A) Transverse section through the intestine of a chub infected with some *C. brachycollis* showing deep penetration of 2 lateral tapeworms (arrows); scale bar = 200 *μ*m. (B) Penetration of tapeworm within the depth of the fold; note spatulate shape of scolex (asterisk) and its intimate contact with the epithelium. Erosion and desquamation (arrows) of the epithelia are evident; scale bar = 200 *μ*m. (C) Micrograph shows interruption of the intestinal muscle layer (asterisk) and disorganization of the epithelia (arrows); *C. brachycollis* (thick arrow) through the hole moves to the chub's body cavity; scale bar = 200 *μ*m. (D) Blanket of mucus (arrows) covers the surface of the epithelium; note the penetration of the scolex (thick arrow) in depth of the fold and numerous mucous cells in contact with parasite tegument; scale bar = 200 *μ*m. (E) Occurrence of blanket of mucus in the interface region between parasite tegument (asterisk) and the epithelium; chalice form mucous cells (arrows) with acid glycoconjugate products are visible; scale bar = 10 *μ*m. (F) Contact between parasite tegument (asterisk) and epithelium; mucous cells (arrows) with mixed glycoconjugate products are evident; scale bar = 10 *μ*m. All the sections were stained with Alcian blue/periodic acid Schiff, AB/PAS.

Examinations of histological sections from the digestive tract of parasitized *S. tenellus* showed the tapeworms penetrated into the deep folds of intestine with scolexes (Fig. 2A and B). The vast majority of the *C. brachycollis* penetrated only the mucosal layer (Fig. 2A and B). The scolex lacked any specialized attachment organs that formed an intimate contact with epithelium which extended around both scolex and neck region (Fig. 2A and B). Furthermore, erosion, desquamation and necrosis of epithelial cells and tissue residues around the worm's body within the intestinal lumen was encountered (Fig. 2B). Nevertheless, sections of the blocks of perforated intestines noticed during chub's necropsy revealed the muscle layers were interrupted by parasite activity-penetration (Fig. 2C). Discharge of mucous cells on the surface of epithelium was more frequent in infected intestines than in uninfected ones; in fish parasitized with tapeworm a blanket of mucus was frequently observed covering the surface of the epithelium (Fig. 2D) and sometimes it was observed at host–parasite interface (Fig. 2E). The current study also investigated the distribution of each type of mucous cell, using their reaction to AB and periodic acid Schiff (PAS) stains to categorize each. In Infected intestine, the mucous cells staining positively for acid glycoconjugates with AB (Fig. 2E) were more abundant in comparison to mixed glycoconjugates (Fig. 2F).

**Figure 3.**
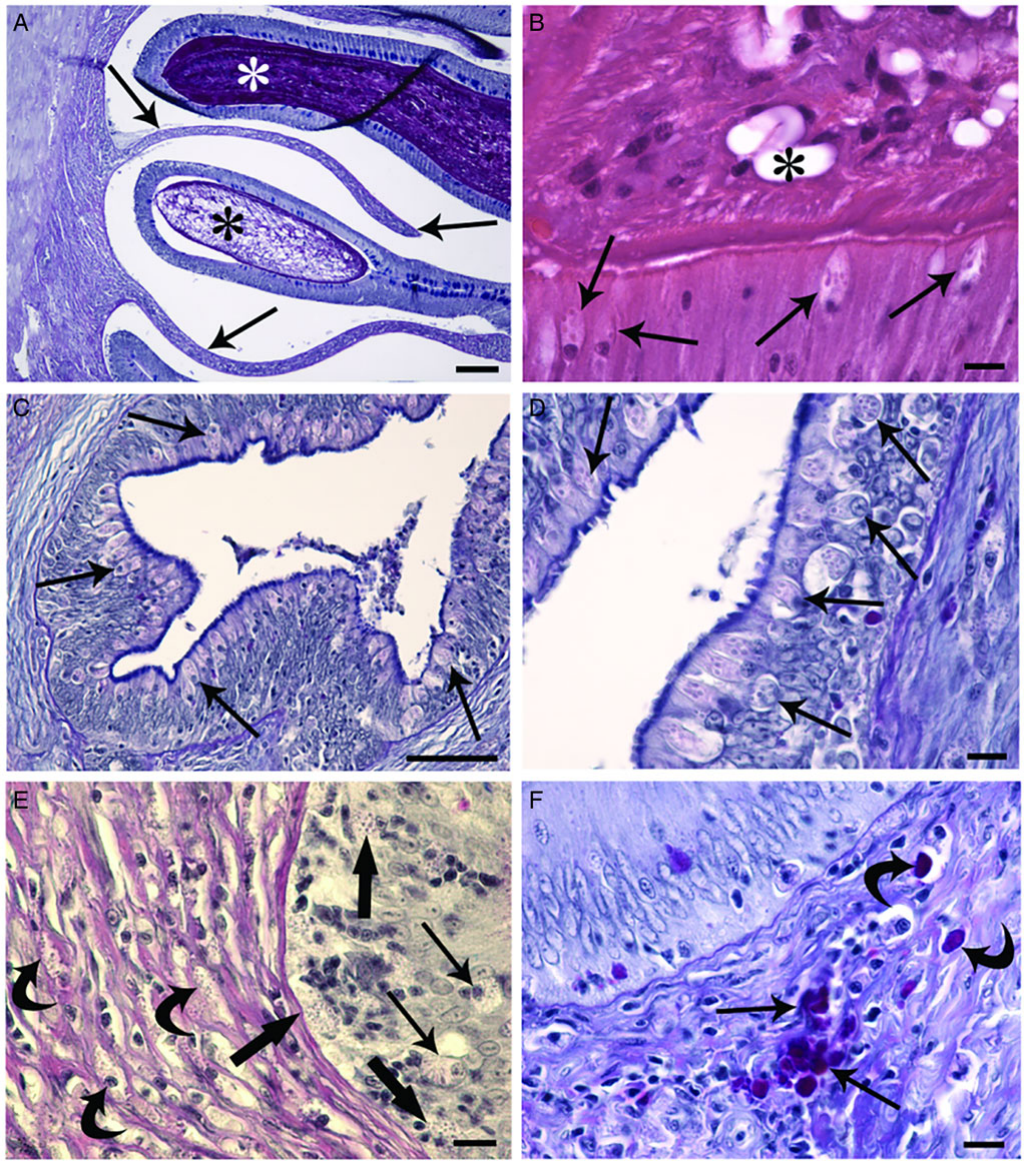
Histological sections of infected intestine of *S. tenellus.* (A) Firm attachment of anterior part of the *C. brachycollis* (asterisks) to the intestine dislodged the epithelium from the folds axes (arrows); scale bar = 100 *μ*m. (B) Tegument of the tapeworm (asterisk) is in contact with the top of the epithelium; some RCs (arrows) are in close proximity to the parasite; scale bar = 10 *μ*m. (C) Epithelium of the infected oesophagus is tapered with numerous RCs (arrows); scale bar = 50 *μ*m. (D) High magnification of the parasitized oesophagus; note the presence of the RCs (arrows) in different levels of the epithelium; scale bar = 10 *μ*m. (E) Micrograph shows MCs (thick arrow) near the RC (arrows) within the epithelium; note numerous neutrophils (curved arrows) in lamina propria-submucosa; scale bar = 10 *μ*m. (F) MAs (arrows) in lamina propria-submucosa of the infected intestine; single macrophage (curved arrows); scale bar = 10 *μ*m. All the sections were stained with Alcian blue/periodic acid Schiff, AB/PAS.

Due to the firm attachment of the anterior part of the *C. brachycollis* to the intestinal wall, often epithelial dislodge from the fold axes was noticed (Fig. 3A). Adjacent to the worm's tegument, among the enterocytes, the presence of several RCs was observed (Fig. 3B). Nonetheless, an impressive number of RCs was documented in the epithelium of the oesophagus (Fig. 3C and D) of some very heavily infected hosts (e.g. chub in Fig. 1B). In some instances, MCs in the epithelium in proximity to the RC were documented (Fig. 3E). Within the tunica propria-submucosa, beneath the site of scolex attachment, numerous neutrophils and very few MCs both cells in intense degranulation and single macrophage and macrophage aggregates (MAs) were noticed (Fig. 3F).

**Figure 4.**
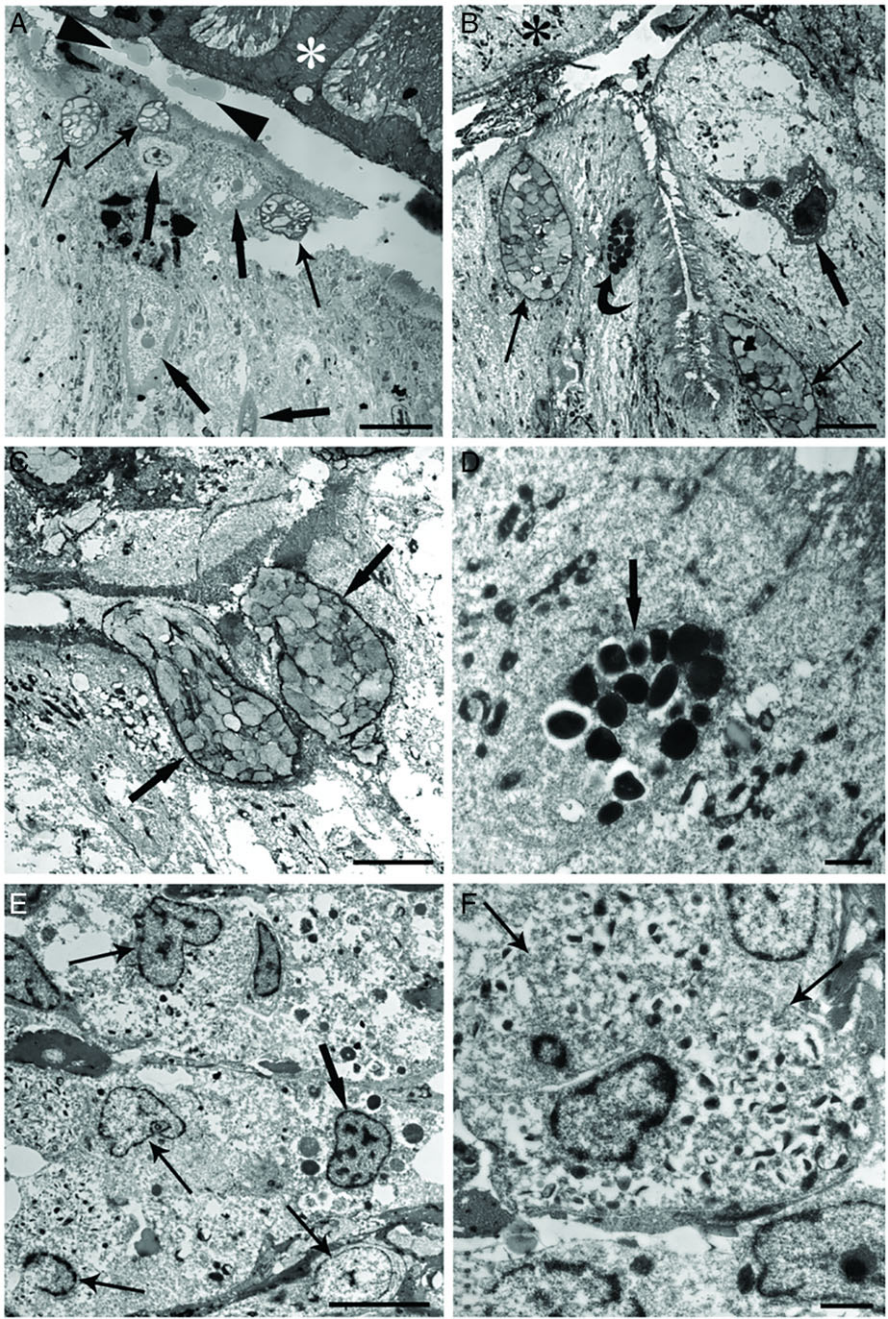
Transmission electron micrographs of interface region between infected intestine of *S. tenellus* and *C. brachycollis* tegument (asterisk). (A) Deformed RCs (thick arrows); mucous cells (arrows); note some vesicles (arrow heads) attached to the parasite's tegument; scale bar = 3 *μ*m. (B) Upper part of the epithelium; deformed RC (thick arrow) with basal heterochromatic nucleus; mucous cells (arrows) with numerous mucous granules and 1 MC (curved arrow) are evident; vacuolation of the enterocytes around the RC is appreciable; asterisk shows parasite tegument; scale bar = 5 *μ*m. (C) Two mucous cells (arrows) released the contents in the lumen; different electron density of mucous granules is visible; vacuolation of the enterocytes around the mucous cells is evident; scale bar = 5 *μ*m. (D) An MC (arrow) in the upper part of the epithelium; note electron-dense aspect of the granules inside the cytoplasm; scale bar = 1 *μ*m. (E) Submucosal layer of the infected intestine; numerous neutrophils (arrows) and 1 MC (thick arrow); both types of cells in intense degranulation; scale bar = 5 *μ*m. (F) Two adjacent neutrophils; note eccentric polar nuclei and rod-shaped electron-dense granules; scale bar = 1 *μ*m.

In TEM sections of the epithelium, in the apical region, near the parasite tegument, mucous cells and RCs were documented (Fig. 4A and B). Figure 4B shows the co-presence of the mucous cells, MCs and RCs in the epithelium not far from the tapeworm body. The MC in the epithelium was oval in shape and contained several electron-dense granules in the cytoplasm (Fig. 4B and D). RCs appeared as pear-shaped cells, and in some instances in proximity to the worm body (Fig. 3A). Each RC had a basal heterochromatic nucleus (Fig. 4B) and cytoplasm filled with few secretory granules called rodlets (Fig. 4A and B). The secretory granules had a unique club-shaped sac with a central core of highly electron-dense material surrounded by less-dense material. In chub intestine, RCs showed moderate-to-intense vacuolization (Fig. 4A), in some instances the cell cortex was deformed (Fig. 4A and B), the vacuolation of the enterocytes was encountered in some infected intestine (Fig. 4B and C).

Concerning the mucous cells, they were chalice form cells (Fig. 2E and F) and scattered among the enterocytes of the intestinal mucosa, with individual cells extending through the simple columnar epithelium from the basal membrane towards the luminal side of the intestine. Commonly, mucous cells documented discharging their contents into the lumen (Fig. 4C). Mucous granules were densely packed into the entire supranuclear cytoplasm (Fig. 4B and C), appearing as spheres or polyhedra surrounded by a single-granule membrane. The mucous granules displayed mainly electron-opaque and, in some cases, as electron-lucent granules (Fig. 4B and C). No noticeable ultrastructural differences were found in intestinal mucous cells in infected/uninfected intestines.

In submucosal sites, beneath the site of attachment of the tapeworm's scolex, numerous neutrophils were noticed; they were irregular in shape with an eccentric polar nucleus and cytoplasm characterized by numerous small, rod-shaped electron-dense granules (Fig. 4E and F). Among neutrophils few MCs were scattered and were frequently encircled by collagen fibres or by fibroblast-like unsheathing cells (not shown). Neutrophils and MCs had intensive vacuolation of the cytoplasm and they frequently were in close proximity or in contact (Fig. 4E). In some grids, we noticed some damaged host cells or their residues in interface region between intestine and tapeworm; indeed, we observed the presence of some vesicles of uncertain origin with amorphous material in close proximity to the *C. brachycollis* body (Fig. 4A).

## Discussion

This investigation is part of a project on alignment of Bosnia and Herzegovina with the European Directives on environmental protection. A subpopulation of a chub *S. tenellus* was examined; this species was introduced to the Lake Blidinje in a homonymous natural park over 100 years ago (Kottelat and Freyhof, 2007). Massive presence of a tapeworm in the intestine of above chub was unexpected (390 worms in a single *S. tenellus*) and lack of information on parasites fauna of *S. tenellus* in the above lake prompted us to undertake this research. In fish, the intensity of infection due to tapeworms often does not exceed 50 parasites/host; nevertheless, in a single common carp, 3000 specimens of a cestode were recorded (Kuchta and Scholz, 2017). The molecular characterization, performed in accordance with Bazsalovicsová *et al*. (2014), based on the sequencing of 2 marker loci, allowed a precise identification of the specimens encountered in *S. tenellus*. This approach appeared more reliable than the morphological analysis alone, as suggested by Nadler and de León (2011), since fish Caryophyllidea species morphologically could show some plasticity (Bazsalovicsová *et al*., 2014; Barčák *et al*., 2017). Our molecular data revealed that the tapeworms belong to *C. brachycollis* species; thus the current study is the first study on type-host (*S. tenellus*) and type-locality (Lake Blidinje, Bosnia-Herzegovina) for this parasite. *Caryophyllaeus brachycollis* was reported in chub *S. cephalus* in a lake in Turkey (Yiğit and Öztürk, 2016); nevertheless, the prevalence (12.3%) and mean intensity (5.3 ± 10 parasites/fish) recorded are much lower than our data (prevalence = 60% and mean intensity, 46.2 ± 15.3 parasites/fish) for the same parasite in chub *S. tenellus*. Concerning *S. tenellus*, there is 1 single record on the occurrence of a monogenean in gills of this chub sampled at Šujica River and Duvansko Poljein in Bosnia-Herzegovina Federation (Benovics *et al*., 2023).

The intestinal canal provides nutrients and protection to helminths (Buchmann, 2014). Extensive literature exists on histopathology caused by helminth parasitism of fish intestine and a recent review of Sayyaf Dezfuli *et al*. (2021*a*, 2023*b*) provides update on this issue. With regards to cestode fish parasites, there have been numerous light investigations on histopathology of order Caryophyllidea (e.g. Mackiewicz *et al*., 1972; Molnár *et al*., 2003; Dezfuli *et al*., 2011; Williams *et al*., 2011) and recently Scholz *et al*. (2021) dealt with histopathology due to different orders of tapeworms and damage they induced to fish. Accurate ultrastructural data on type of host cells involved in response to fish intestinal tapeworms are limited and include (Karanis and Taraschewski, 1993; Hoole and Nisan, 1994; Morley and Hoole, 1995; Sayyaf Dezfuli *et al*., 2021*a*). Reports that tapeworms cause fish mortalities are very few (e.g. Scott and Grizzle, 1979); accordingly, *Ligula intestinalis* (Cestoda) can induce mortality of fish (second intermediate host) either directly from fish inability to survive through winter (Wyatt and Kennedy, 1989) and indirectly through increased predation risk by birds or other fish (Palm *et al*., 2018). It seems that, cestodes have relatively minor impact on farmed fish in comparison to the effect of other pathogens/parasites (Shinn *et al*., 2015).

The intestine is a particularly competitive ecological environment; infection of the alimentary canal by helminths has detrimental effects on digestive function; intestinal inhabitants have found ways to utilize the host to achieve a competitive advantage in this nutrient-rich environment (Loke and Harris, 2023). Attachment organ of endoparasitic worms often induces intense inflammation (Karanis and Taraschewski, 1993; Williams *et al*., 2011; Sayyaf Dezfuli *et al*., 2021*a*, 2022). Inflammation is the host response to invasion by foreign organisms as well as to physical injury and serves to protect the host by evoking specific chemical and morphological alterations to the injured tissues (Johansson and Hansson, 2014; Birchenough *et al*., 2015). The long lifespan of enteric helminths creates chronic infections and the initial immune response mounted by hosts often progresses into a chronic condition characterized by pathological changes to the gut tissue (Wanstall *et al*., 1986; Karanis and Taraschewski, 1993; Sayyaf Dezfuli *et al*., 2021*a*).

It is generally accepted that the pathogenicity of most enteric helminths is attributed to 2 factors: density of the parasite burden and depth of worm penetration (Mackiewicz *et al*., 1972; Bosi *et al*., 2022). Several species of cestode do not penetrate deeply into the fish intestinal layers; the main damage caused by them is the destruction of the mucosal epithelium covering the villi with consequent necrosis and degeneration, mild inflammation and excessive secretion of the mucus (Mackiewicz *et al*., 1972; Dezfuli *et al*., 2010; Santos *et al*., 2017; Barčák *et al*., 2021; Kumari and Nomani, 2022). Conversely, some other tapeworm species provoke total destruction of the lamina mucosa and lamina submucosa, reaching the muscular layer and inducing an intense inflammatory response in intestine (Morley and Hoole, 1995; Dezfuli *et al*., 2011; Williams *et al*., 2011; Barčák *et al*., 2021; Scholz *et al*., 2021). Herein, in 3 very infected chubs, *C. brachycollis* perforated the entire intestinal wall (Figs 1 and 2C) and their strobilae were free in the body cavity. We are not aware of other tapeworm species which punctures intestine of fish definitive host; the case of *L. intestinalis* is different, this cestode inhabits in the body cavity of fish (second intermediate host) until predation by piscivorous birds (definitive host) (Palm *et al*., 2018).

Numerous studies have documented that the presence and action of the enteric helminths in fish recruit different types of inflammatory cells belong to the innate immune system, as well as a network of nervous fibres at the site of infection (Dezfuli *et al*., 2016; de Sales-Ribeiro *et al*., 2021; Bosi *et al*., 2022). Herein, in the *S. tenellus*–*C. brachycollis* system occurrence of mucous cells, RCs, MCs, neutrophils and MAs was documented. Below, we will examine in turn each above cell type involved in the response of chub intestine to the tapeworm.

Concerning the mucous cells, in the alimentary canal of vertebrates, the epithelial surface is protected by a mucus blanket/barrier made up of the polymeric mucins secreted by mucous cells. Although the function of mucus has historically been suggested to act only as a physical barrier, it is now accepted that it has other general intrinsic roles such as lubrication, hydration, providing specific ligands for pathogen entrapment and helping digestion (Corfield *et al*., 2000). Mucins are high molecular weight, glycosylated proteins (Schroers *et al*., 2009) and are important elements for initial protection against enteric helminths (Sharpe *et al*., 2018; Bosi *et al*., 2020; Sayyaf Dezfuli *et al*., 2021*a*). In fish, the intestinal mucosal surface is constantly exposed to numerous microorganisms and foreign substances from ingested water (Neuhaus *et al*., 2007). The secretion of intestinal mucins increases under pathological conditions, as showed by the hyperplasia and hypertrophy of the mucous cells in several fish–helminth systems (Dama and Pathan, 2019; Souza *et al*., 2019; Sayyaf Dezfuli *et al*., 2021*a*; Scholz *et al*., 2021; Bosi *et al*., 2022). The tissue damage caused by helminth infections induces rapid production of cytokines and chemokines by innate immunity cells, such as type 2 innate lymphoid cells, with mobilization of neutrophils, basophils and eosinophils (Harris and Loke, 2017). During the observations of the histological slides of the intestine of *S. tenellus*-harboured *C. brachycollis*, we noticed that the mucous cells containing acidic glycoconjugates were more than cells with mixed glycoconjugates. The same finding was reported in other studies (Dama and Pathan, 2019; Bosi *et al*., 2020). Accordingly, an increase in acidic mucins, which is associated with an increased viscosity of secreted mucus, provides enhanced protection against pathogens–parasites (Tibbets, 1997; Díaz *et al*., 2008; Bosi *et al*., 2017).

RCs are pear-shaped cells characterized by a distinctive cortex, basal nucleus and conspicuous typical inclusions called rodlets (Reite and Evensen, 2006; Bosi *et al*., 2018). RCs are primarily found in the epithelial tissue of different organs of freshwater and marine fish (Sayyaf Dezfuli *et al*., 2022). Some studies suggest that RCs are a type of inflammatory cell closely associated with other piscine inflammatory cells, such as MCs, mesothelial and epithelioid cells (Reite and Evensen, 2006). Indeed, RCs are considered a kind of secretory cell and proliferate in response to tissue injury or related factors (Leino, 1996). In the intestines of some fish species, RCs express immune molecular markers, including lysozyme and *α*-*N*-acetyl-galactosamine (Bosi *et al*., 2018). Records concerning the role of RCs as immune effector cells have focused on their mobilization and recruitment in response to microparasites (Salinas *et al*., 2008; Sitjà-Bobadilla *et al*., 2016). In fish-harboured macroparasites, the occurrence of high number of RCs, particularly at the site of parasite attachment, provides further evidence of their defensive function as part of the innate immune system (Reite, 2005; Matisz *et al*., 2010; Sayyaf Dezfuli *et al*., 2022, 2023*a*, 2023*b*). Herein, occurrence of the RCs was noticed in the intestine of infected chub and in close vicinity to the *C. brachycollis* tegument, and it was surprising to see numerous RCs scattered within the epithelium of very infected oesophagus. Previously, cluster of the RCs was noticed in the intestine of eel *Anguilla anguilla* infected with unknown bacteria in lumen (Bosi *et al*., 2017) and eel gut parasitized with a coccidian (Sayyaf Dezfuli *et al*., 2023*a*).

MCs exist in all classes of vertebrates, and share similar morphology and function (Mulero *et al*., 2008; Baccari *et al*., 2011); they are a type of less mobile tissue granulocytes (Reite and Evensen, 2006; Sayyaf Dezfuli *et al*., 2020, 2023*b*). Fish MCs are irregular in shape and cytoplasm is filled with numerous large, electron-dense granules; these cells are strategically positioned at perivascular sites to regulate inflammation and coordinate an adequate response (John and Abraham, 2013). MCs react to parasite exposure by releasing their contents through degranulation, a process that has been frequently documented in fish infected with metazoan parasites (Sayyaf Dezfuli *et al*., 2021*a*). MC granules contain a panel of inflammation mediators including piscidins (Silphaduang *et al*., 2006; Dezfuli *et al*., 2010; Salger *et al*., 2016), serotonin (Dezfuli *et al*., 2000; Da Silva *et al*., 2017), mucopolysaccharides with residues of *α*-*N*-acetyl-galactosamine (Dezfuli *et al*., 2015) and finally, histamines (Mulero *et al*., 2008; Salim *et al*., 2012; Galindo-Villegas *et al*., 2016; Sayyaf Dezfuli *et al*., 2018). It is well known that, most parasitic helminths due to their attachment structures and/or penetration in host organ induce damages, and one of the functions of the MCs is tissue repair and remodelling (Dezfuli *et al*., 2015). The occurrence of proliferating cell nuclear antigen (PCNA) demonstrates an increase in the rate of cell division in tissues (Ortego *et al*., 1994). Significant increase in the number of PCNA-positive MCs at the site of helminth infection was documented in some papers (e.g. Dezfuli *et al*., 2015). Several records mentioned that fish possess both a local and a circulating MC population, with parasitism inducing recruitment of MCs to the site of infection (Alvarez-Pellitero, 2011; Sayyaf Dezfuli *et al*., 2018) and proliferation of the local population of the MCs (Sayyaf Dezfuli *et al*., 2020). Herein, in epithelium and in tunica propria-submucosa below the site of the attachment of *C. brachycollis* the presence of some MCs was documented. Acute MC activation-recruitment is a feature of several types of tissue injury and occurrence of parasites (Sayyaf Dezfuli *et al*., 2021*a*, 2021*b*); moreover, experimental studies have shown that pathogen products can also activate MCs (Flaño *et al*., 1996).

In fish, 2 major phagocyte populations are: granulocytes (particularly neutrophils) and mononuclear phagocytes (circulating monocytes and tissue macrophages) (Secombes, 1996). With reference to the neutrophils, they are among the first cell types to arrive at the site of infection or inflamed tissue (Amulic *et al*., 2012; Bader *et al*., 2021). In fish neutrophils account for ~5% of circulating leucocytes (Jørgensen *et al*., 2018), whereas in mammals they represent the predominant leucocytes during homoeostasis. Kidney of teleost as haematopoietic organ has the largest population of neutrophils, which can be rapidly mobilized through blood vessels to sites of inflammation (Havixbeck *et al*., 2016; Fingerhut *et al*., 2020). In fish as in mammals, the chemokine interleukin-8 (also known as CXCL8) is involved in recruiting neutrophils to the site of infection (de Oliveira *et al*., 2015).

In addition to phagocytosis, neutrophils secrete active molecules and radicals (Neumann *et al*., 2001); these reactive substances exert biocidal actions against bacteria and parasites and are involved in cytokine responses and modulation of immune cell apoptosis (Katzenback and Belosevic, 2012). The cytoplasmic granules of neutrophils contain mainly myeloperoxidase, a highly cationic glycosylated enzyme primarily produced by these leucocytes (Secombes and Ellis, 2012; Havixbeck and Barreda, 2015). Neutrophils also contribute to pro-inflammatory responses by releasing cytokines that activate and recruit other host immune cells (Harvie and Huttenlocher, 2015*a*, 2015*b*). The relationship between neutrophils and aquatic pathogens has been recently reviewed by Buchmann (2022); records on enteric helminths–neutrophils were provided in review of Sayyaf Dezfuli *et al*. (2021*a*). Neutrophils as highly motile cells play a crucial role in the initial defence through phagocytosis of microbes, secretion of granule proteins and release of other antimicrobials (Harvie and Huttenlocher, 2015*a*, 2015*b*). Herein, in lamina propria of chub intestine, below the scolex of *C. brachycollis* the massive presence of the neutrophils was documented and most of them were in intense degranulation. Such aspect of neutrophils and big size of the *C. brachycollis* might suggest that infection in that site was not recent and fish faced a chronic inflammation.

The primary phagocytic cells in vertebrates are macrophages and their precursor monocytes, macrophages are key innate immune cells that respond to tissue-environment alterations (Harris and Loke, 2017). Macrophage-lineage cells are crucial to bridge the innate and adaptive arms of the vertebrate immune response (Grayfer *et al*., 2018). In response to inflamed tissue and infection caused by parasitic pathogens, monocytes are promptly recruited and undergo differentiation into tissue macrophages (Grayfer *et al*., 2018). Fish macrophages are found in kidney, liver, spleen, intestine, gills and in the body cavity (Secombes and Ellis, 2012). Macrophages are characterized as large cells with an irregular outline and often contain pigments like lipofuscin, haemosiderin and melanin (Agius and Roberts, 2003; Secombes and Ellis, 2012; Stosik *et al*., 2019) and can be organized in groups known as melano-macrophage centres or MAs (Agius and Roberts, 2003; Stosik *et al*., 2019; Sayyaf Dezfuli *et al*., 2021*b*). Recent studies have reported the presence of resident macrophage populations in various tissues, which exhibit rapid and highly specific responses to pathogen (Shapouri-Moghaddam *et al*., 2018; Lu and Chen, 2019; Graves *et al*., 2021).

In response to signals from the surrounding tissues, macrophages undergo molecular changes and exhibit different functional behaviours through a process known as macrophage polarization, such task likely is due to pathogens or their excreted–secreted molecules (Arango and Descoteaux, 2014; Earley *et al*., 2018; Lu and Chen, 2019; Wiegertjes and Elks, 2022). Some records on response of fish macrophages and MAs against helminth infections appeared in Whyte *et al*. (1989) and Sayyaf Dezfuli *et al*. (2021*a*, 2021*b*). At the site of inflammation, macrophages are exposed to pro-inflammatory stimuli and dying cells (Rieger *et al*., 2012). It is known that the intestine contains the largest pool of macrophages, responsible for epithelial renewal and mucosal homoeostasis maintaining (Rieger *et al*., 2012; Grayfer *et al*., 2018). Macrophages appear to be maintained in a steady state within the lamina propria of the fish intestine, protecting the mucosa against parasites and engulfing pathogens and the debris of damaged cells (Sitjà-Bobadilla *et al*., 2016; Sayyaf Dezfuli *et al*., 2021*a*). Neutrophil-derived LTB4 induces macrophage aggregation formation (Vincent *et al*., 2017). Our results on occurrence of the MAs in parasitized intestine of *S. tenellus* tally with several studies which justify the occurrence of macrophages and MAs in fish-infected organs (Molnár, 2005; Mulero *et al*., 2008; Estensoro *et al*., 2014; Sayyaf Dezfuli *et al*., 2020).

In some grids, in interface region between intestine–*C. brachycollis* tegument residues of host damaged cells were noticed; the same finding was reported in tench intestine heavily infected with tapeworm *Monobothrium wageneri* (Dezfuli *et al*., 2011). In the interface region, the presence of some vesicles filled with amorphous material was observed; further investigations are needed before any speculation on their origin and nature of the content.

All fish specimens examined in this study were alive, active and had normal colour, and upon necropsy, the liver in infected/uninfected chubs presented the normal reddish colour with no evident clinical signs. Moreover, examination of liver histological sections showed that the hepatic tissue had homogeneous parenchyma and normal distribution of the melano-macrophage centres. According to Noga (2010), an organ with these features can be considered a liver without remarkable pathology. Nevertheless, we had only 21 infected chubs; before providing any conclusion with confidence on the effect of tapeworm on the condition factor of chub, more infected *S. tenellus* are necessary.

## Data Availability

Not applicable.
